# Alzheimer’s disease alters the transcriptomic profile of natural killer cells at single-cell resolution

**DOI:** 10.3389/fimmu.2022.1004885

**Published:** 2022-11-02

**Authors:** Caiyun Qi, Fang Liu, Wenjun Zhang, Yali Han, Nan Zhang, Qiang Liu, Handong Li

**Affiliations:** Department of Neurology, Tianjin Neurological Institute, Tianjin Medical University General Hospital, Tianjin, China

**Keywords:** Alzheimer’s disease, natural killer cell, single cell sequencing, cytotoxicity, innate immunity

## Abstract

Alzheimer’s disease (AD) is the most common dementia without an effective cure at least partially due to incomplete understanding of the disease. Inflammation has emerged as a central player in the onset and progression of AD. As innate lymphoid cells, natural killer (NK) cells orchestrate the initiation and evolution of inflammatory responses. Yet, the transcriptomic features of NK cells in AD remain poorly understood. We assessed the diversity of NK cells using web-based single-cell RNA sequencing data of blood NK cells from patients with AD and control subjects and flow cytometry. We identified a contraction of NK cell compartment in AD, accompanied by a reduction of cytotoxicity. Unbiased clustering revealed four subsets of NK cells in AD, i.e., CD56^bright^ NK cells, CD56^dim^ effector NK cells, adaptive NK cells, and a unique NK cell subset that is expanded and characterized by upregulation of CX3CR1, TBX21, MYOM2, DUSP1, and ZFP36L2, and negatively correlated with cognitive function in AD patients. Pseudo-temporal analysis revealed that this unique NK cell subset was at a late stage of NK cell development and enriched with transcription factors TBX21, NFATC2, and SMAD3. Together, our study identified a distinct NK cell subset and its potential involvement in AD.

## Introduction

Alzheimer’s disease (AD) is the most common dementia type with limited therapeutic options partially due to the incomplete understanding of disease. Inflammation has emerged as a major contributor to AD pathogenesis (1–3). Mounting evidence has demonstrated that microglia and hematogenous myeloid cells participate in β-amyloid pathology and cognitive decline (4–6). In contrast, the involvement of lymphocytes in the etiology of AD is less studied.

As innate lymphoid cells, natural killer (NK) cells are critical players that control the initiation and progression of brain inflammation (7, 8). NK cells are generally divided into CD56^bright^ and CD56^dim^ subsets. As a less numerous subset, CD56^bright^ cells are the primary source of NK cell-derived regulatory cytokines, whereas CD56^dim^ cells are mainly cytotoxic effector cells producing IFN-γ upon stimulation. However, the phenotype and function of NK cells in AD remain poorly understood. To address this question, we assessed the transcriptomic alterations of NK cells in AD by analyzing web-based single-cell RNA sequencing data of blood NK cells. As a result, we found reduced number and cytotoxic activity of blood NK cells in AD patients versus control subjects. In particular, we identified an increase of a unique NK cell subset that is at a late stage of development and enriched with transcription factors (TFs) TBX21, NFATC2 and SMAD3, and negatively correlated with the cognitive decline in AD.

## Materials and methods

### Single-cell RNA sequencing data collection

Single-cell RNA sequencing data of 36,830 cells from a recent published study on human peripheral blood mononuclear cells (PBMCs) from three patients with AD (two men and one woman, 22,770 cells) and two control subjects (one man and one woman, 14,060 cells) were accessed from the GEO public database (GSE181279) (9). There was no significant statistical difference regarding the age between two groups (AD vs. control: 67.7 ± 8.6 vs. 71.0 ± 8.5 years, *p* = 0.699). Low-quality cells with <200 genes, >20,000 UMI, and >10% mitochondrial genes as well as genes that expressed less than three cells were filtered out. The remaining 36,561 cells were finally included in the analysis.

### Data integration, dimensionality reduction, clustering, and visualization

Seurat (v 4.1.0) (10) was used for dimensionality reduction, clustering, and visualization. For each sample dataset, we used the filtered expression matrix to identify cell subsets. The filtered gene expression matrix was normalized using the NormalizeData function, in which the number of UMIs of each gene was divided by the sum of the total UMIs per cell, multiplied by 10,000, and then transformed to log scale (in UMI-per-10,000+1). After normalization, the data were scaled with the ScaleData function, and the top 2,000 highly variable genes were identified by the FindVariableFeatures function and used for the following principal component analysis (PCA). Subsequently, the harmony v1 integration method was used to correct the potential batch effect and then clustering with top 20 principal components and resolution 0.5 was performed by graph-based clustering and visualized using t-Distributed Stochastic Neighbor Embedding (t-SNE) with Seurat functions RunTSNE. After the identification of cell types, NK cells were extracted and subclustered for further detailed analysis. The subclustering was performed *via* Seurat with top 13 principal components and a resolution of 0.4. To identify cell types in sample datasets, we used sets of marker genes for each of those cell types and annotated each cell type based on their average expression and expression ratio as previously described (11, 12).

### Differential gene expression analysis

Differentially expressed genes (DEGs) in a given cell type compared with all other cell types were determined with the FindAllMarkers function from the Seurat package (Wilcoxon rank-sum test, *p*-values adjusted for multiple testing using Bonferroni correction). The FindMarkers function was used to compute the DEGs between groups. We set min.pct = 0 and logfc.threshold = 0 to obtain all the DEGs and finally filter by *p*-value < 0.05 to draw the DEGs’ volcano plot.

### Enrichment analyses of differentially expressed genes

The enrichGO and enrichKEGG (cutFC = 0.5) functions of the RNAseqStat R (https://github.com/xiayh17/RNAseqStat) package were used to calculate and visualize the enrichment results of the whole NK cells’ DEGs between AD and controls; the Gene Ontology (GO) enrichment mainly displayed the enrichment results of upregulated genes. The Database for Annotation, Visualization, and Integrated Discovery (DAVID, https://david.ncifcrf.gov/) was used to annotate and analyze the associated GO terms and Kyoto Encyclopedia of Genes and Genomes (KEGG) and Reactome pathways of the DEGs (*p*-value < 0.05 and LogFoldChange > 0.25). GO terms and KEGG and Reactome pathways with adjusted *p*-value < 0.05 were considered significant.

### Gene set module score analyses

To further verify the identity of each NK cell cluster, the AddModuleScore function was used to calculate the gene set score as previously defined (13–16): (i) blood CD56^dim^ NK (FGFBP2, GZMB, GZMA, SPON2, S100A4, CST7, FCGR3A, IGFBP7, GZMH, and CFL1); (ii) blood CD56^bright^ NK (GZMK, CD44, PPP1R14B, CXCR3, RPL36A, SCML1, COTL1, NCF1, XCL1, and HLA-DRB1); and (iii) adaptive NK (KLRC2, CD52, IL32, CD3E, CD3D, CD3G, B3GAT1, TTC16, CADM1, SGCD, VIM, and CCL5). Owing to the similarity between NK and ILC1 cells, we identified ILC1 by scoring the gene signatures of CXCR3, IFNG, LTA, IL12RB1, TBX21, IKZF3, LEF1, ZBP1, JUNB, TSHZ2, SP140, BCL11B, PRDM1, IL6R, IL6ST, IL18BP, SOCS3, IFNG-AS1, GZMM, GZMK, GZMA, SH2D1A, CD6, CD27, CD5, CCR7, CD28, TNFRSF1B, TNFSF8, TNFRSF10A, CCL5, LAG3, CD3D, CD3E, CD3G, CD4, CD8A, CD8B, TRAV13-1, TRAV8-2, TRAV4, TRBV5-1, TRAV9-2, TRAV2, TRBV2, TRAV41, TRBV20-1, TRAV26-2, and TRAV8-4 as previously described (17). Cell subclusters with high score were deemed as ILC1 and excluded.

### Pseudo-time analysis

For NK cell subclusters, we performed pseudo-time analysis with Monocle2 (v2.18.0). The ordering was based on the 3,801 DEGs between clusters. Then, the data space was reduced by DDRTree algorithm into two dimensions. The cells were finally ordered in pseudo-time and clusters with a high score of CD56^bright^ NK signatures were considered as the start point of the trajectory.

To identify significantly branch-dependent genes, we used the BEAM algorithm function and gene significance was set to *q*-value < 1E-04. The selection of branch-dependent TFs was according to the intersection between 682 branch-dependent genes and human TF sets from the Human TFDB database.

### Protein–protein interaction network construction

After the identification of TFs, STRING (https://cn.string-db.org/) was used to construct the protein–protein interaction network.

### Human peripheral blood samples

Peripheral blood samples were collected from seven patients with AD (three men and four women) and 11 control subjects (six men and five women). There was no statistically significant difference regarding the age (AD vs. Control: 61.6 ± 7.8 vs. 66.5 ± 6.7, *p* = 0.202) and sex (*p* = 0.280) of AD patients vs. control subjects. For AD patients, participants were diagnosed with AD (IWG-2) and had positive amyloid PET imaging. Control subjects were generally healthy with normal laboratory test results.

### Flow cytometry

PBMCs were isolated from whole-blood specimens and stained with fluorescent-labeled antibodies. For the staining of the intracellular molecules, cells were fixed and permeabilized using commercial kit (eBioscience) according to the manufacturer’s instruction. All antibodies were purchased from BioLegend (San Diego, CA, USA), including CD3 (UCHT1), CD56 (HCD56), CD69 (FN50), CD27 (O323), NKG2D (1D11), CX3CR1 (2A9-1), and IFN-γ (4S.B3). Flow cytometry was performed using FACS Aria III (BD Bioscience, San Jose, CA, USA). Data were all analyzed by FlowJo v10.8.1.

### Statistical analysis

All statistical analyses of single-cell sequencing data were performed using R software, version 4.1.3. Data represent mean ± SEM. The statistical significance of module gene set analysis was assessed by the Wilcoxon rank sum test with continuity correction or by the Kruskal–Wallis test with Dunn’s multiple comparisons test, with *p*-value adjustment by the Benjamini–Hochberg method. Unpaired Student’s t test was employed to compare flow cytometry data from AD patients in comparison to controls. *p* < 0.05 was considered to be statistically significant.

## Results

### Single cell transcriptomic analysis of PBMCs from patients with AD

**Figure 1:**
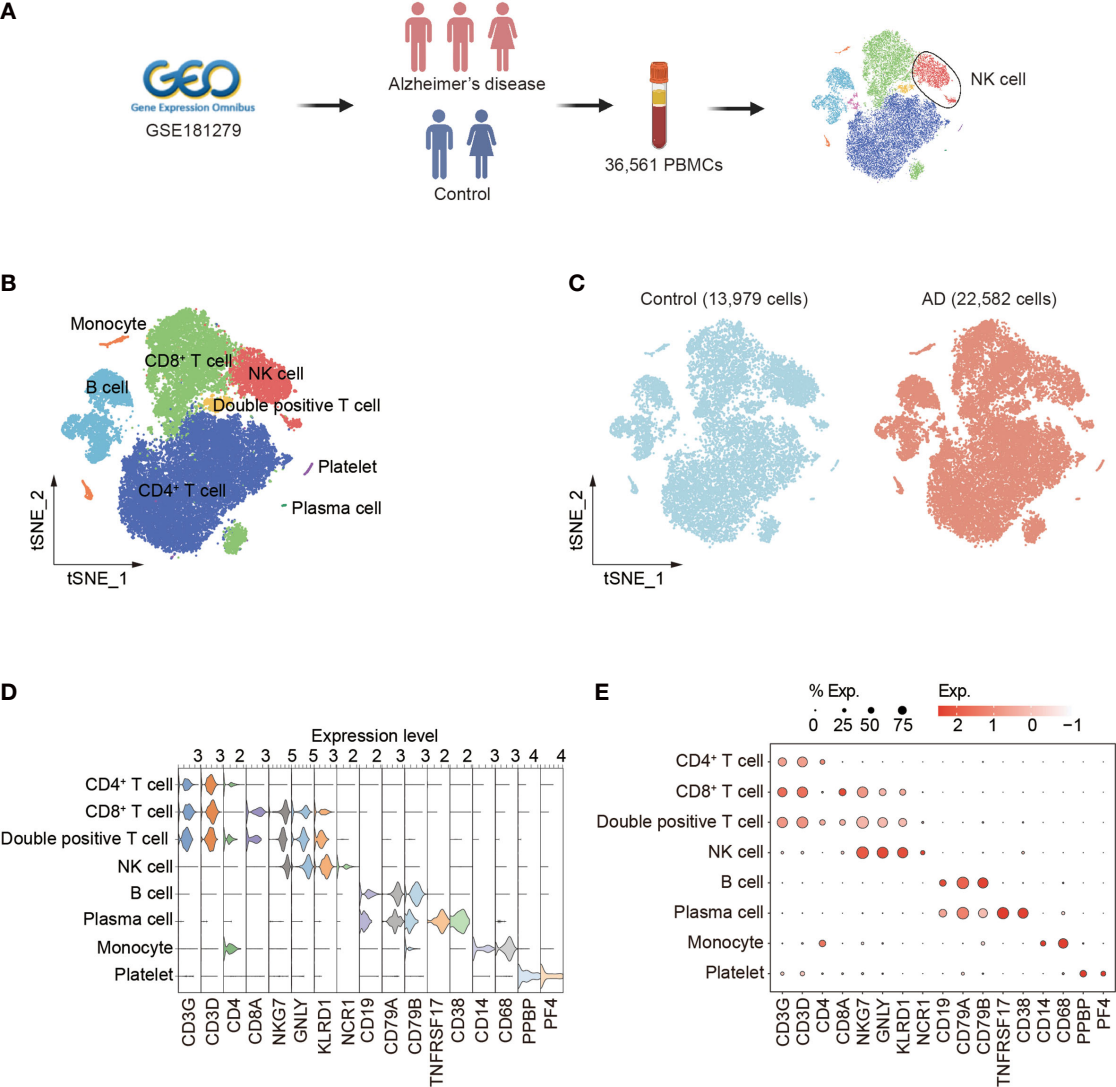
Single-cell transcriptomic analysis of PBMCs from patients with AD. **(A)** Schematic of experimental design. **(B)** tSNE plot of 36,561 single cells from total PBMCs of three AD patients and two control subjects. **(C)** tSNE plots of 13,979 single cells from control subjects and 22,582 single cells from AD patients, colored by group. **(D, E)** The violin **(D)** and dot **(E)** plots showed expression levels of known cell type-specific markers. PBMCs: peripheral blood mononuclear cells; tSNE: t-distributed Stochastic Neighbor Embedding.

We analyzed the single-cell RNA-sequencing data of PBMCs (GEO database: GSE181279) from three patients with AD and two control subjects. A total of 36,561 cells were included for assessment. Among these cells, 22,582 were from AD patients and 13,979 were from control subjects (**Figure 1A**, **Supplementary Figures 1A–C**). Unbiased clustering identified eight major cell subsets based on specific markers: CD4^+^ T cells (CD3D, CD3G, and CD4), CD8^+^ T cells (CD3D, CD3G, and CD8A), double-positive T cells (CD3D, CD3G, CD4, and CD8A), NK cells (identified as expression of NKG7, KLRD1, and NCR1, and lack of expression of CD3), B cells (CD19, CD79A, and CD79B), plasma cells (CD19, CD79A, TNFRSF17, and CD38), monocytes (CD14), and platelets (PPBP and PF4) (**Figure 1B**). The cellular distribution of each group is shown in **Figure 1C**. The dot and violin plots displayed the expression of specific genes in each cluster (**Figures 1D, E**).

### Reduced number and cytotoxicity in blood NK cells from patients with AD

**Figure 2:**
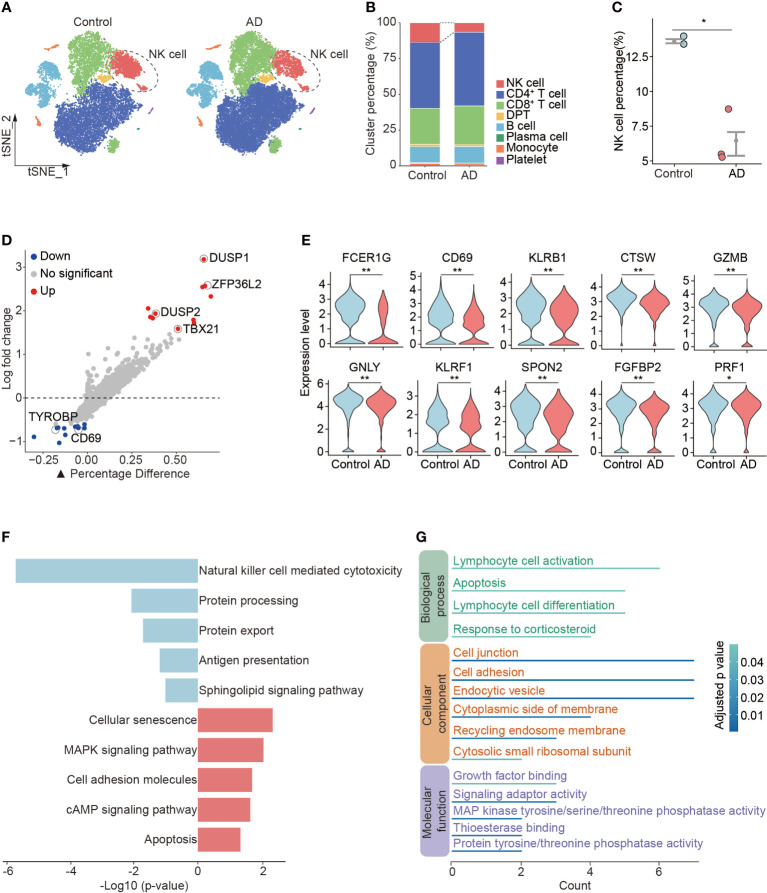
Reduced number and cytotoxicity in blood NK cells from patients with AD. **(A)** tSNE plots of total PBMCs from controls and patients with AD. NK cells were labeled with ellipse tag, which were reduced in the AD group. **(B)** The distribution of cell clusters in AD and control groups. **(C)** NK cell percentage in individual level. Data are presented as means ± SEM. *p < 0.05, **p < 0.01. **(D)** Assessment of differentially expressed genes (DEGs) using log-fold change expression versus the difference in the percentage of cells expressing the gene in blood NK cells from patients with AD versus controls (▲Percentage Difference). Genes labeled were chosen based on log-fold change > 1.5 (Up) and log-fold change < −0.5 (Down), adjusted *p*-value from Wilcoxon rank sum test < 0.05. (**E**) Violin plots show the expression levels of cytotoxicity and activation markers in blood NK cells from patients with AD versus controls. **(F)** KEGG pathway analysis of DEGs in blood NK cells from patients with AD versus controls. Blue: downregulated pathways, pink: upregulated pathways. **(G)** GO enrichment analysis of DEGs in NK cells.

In the determination of cellular composition and distribution, we found a reduction of blood NK cells in patients with AD versus control subjects (**Figures 2A–C**, **Supplementary Figure 1D**). NK cells from patients with AD displayed upregulation of DUSP1 and DUSP2 that are regulators of the ERK signaling pathway and the RNA-binding protein ZFP36L2 that is related to immunosuppression as well as TBX21 involved in NK cell maturation (**Figure 2D**). We also observed a decrease in cytotoxicity genes (FCER1G, CTSW, GZMB, GNLY, KLRF1, SPON2, FGFBP2, and PRF1) and activation markers (CD69 and KLRB1) in NK cells from patients with AD (**Figure 2E**). The KEGG analysis also revealed a reduction of NK cell-mediated cytotoxicity (**Figure 2F**). GO analysis revealed the DEGs in NK cells regarding lymphocyte activation, cell adhesion, and related intracellular pathways (**Figure 2G**).

To verify the above findings, we conducted flow cytometry analysis of peripheral blood from 7 AD patients and 11 control subjects. In line with scRNA-seq results, we found a reduction of NK cell number and percentage in peripheral blood from AD patients, though the difference was not significant (**Supplementary Figures 2A, B, D**). We also found reduced expression of CD69, CD27, NKG2D, and IFN-γ in NK cells from peripheral blood of AD patients (**Supplementary Figures 2C, D**). Together, these results suggest reduced number and cytotoxicity in blood NK cells from patients with AD.

### Subclustering analysis revealed expansion of a unique NK cell subset expressing CX3CR1, TBX21, MYOM2, DUSP1, and ZFP36L2 in patients with AD

**Figure 3:**
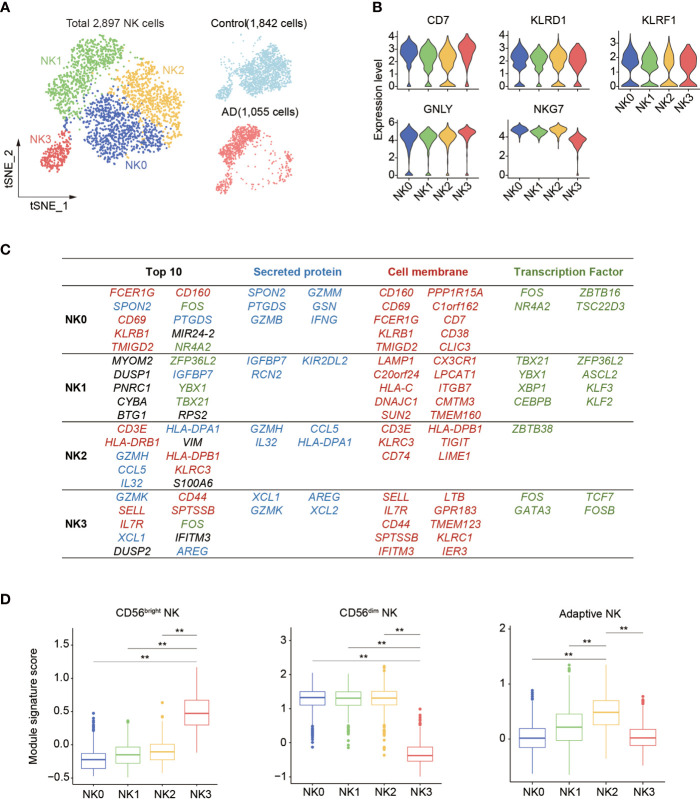
Subclustering of blood NK cell in patients with AD. **(A)** tSNE plot of 2,897 NK cells from three patients with AD and two control subjects. **(B)** Violin plots display the expression of NK cell lineage markers in each cluster. The *y*-axis represents normalized expression value. **(C)** Top 10 most enriched genes among the total gene set and among upregulated genes encoding secreted proteins, cell membrane markers, and transcription factors that are different among four NK cell subpopulations. Blue: secreted protein, red: cell membrane, green: transcription factors. **(D)** The boxplots showing the distribution of the module score for blood CD56^dim^ as well as CD56^bright^ NK cells and adaptive NK cells among each NK cell subset. **p < 0.01.

Next, subclustering analysis was performed to assess the transcriptomic alterations in blood NK cells from patients with AD after exclusion of ILCs *via* module score analysis. Unsupervised clustering of the remaining 2,897 NK cells (Control: 1,842 cells, AD: 1,055 cells) revealed four subsets: NK0, NK1, NK2, and NK3 (**Figure 3A**, **Supplementary Figure 1E**). As shown in **Figure 3B**, the expression of NK cell signatures (CD7, NKG7, GNLY, KLRD1, and KLRF1) was identified in these subsets.

Thereafter, we assessed the top genes that were expressed among these four NK cell subsets (**Figure 3C**). We found that the NK0 cluster expressed genes associated with cytotoxic factors (FCER1G, SPON2, GZMM, and GZMB) and activation markers CD69 and CD160, resembling CD56^dim^ effector NK cells. The NK1 cluster displayed an enrichment of genes including CX3CR1, TBX21, MYOM2, DUSP1, and ZFP36L2. The expression of CX3CR1, TBX21, and KIR2DL2 suggests that the NK1 subset was in the late stage of NK cell development as previously described (14). The NK2 cluster expressed CD3E, GZMH, CCL5, IL32, VIM, and KLRC3 as well as HLA molecule-encoding genes (HLA-DRB1, HLA-DPB1, and HLA-DPA1) related to previously reported adaptive NK cells (14, 15). The NK3 cluster expressed GZMK, IL7R, SELL, XCL1, XCL2, KLRC1, and CD44, resembling CD56^bright^ NK cells (13–15).

**Figure 4:**
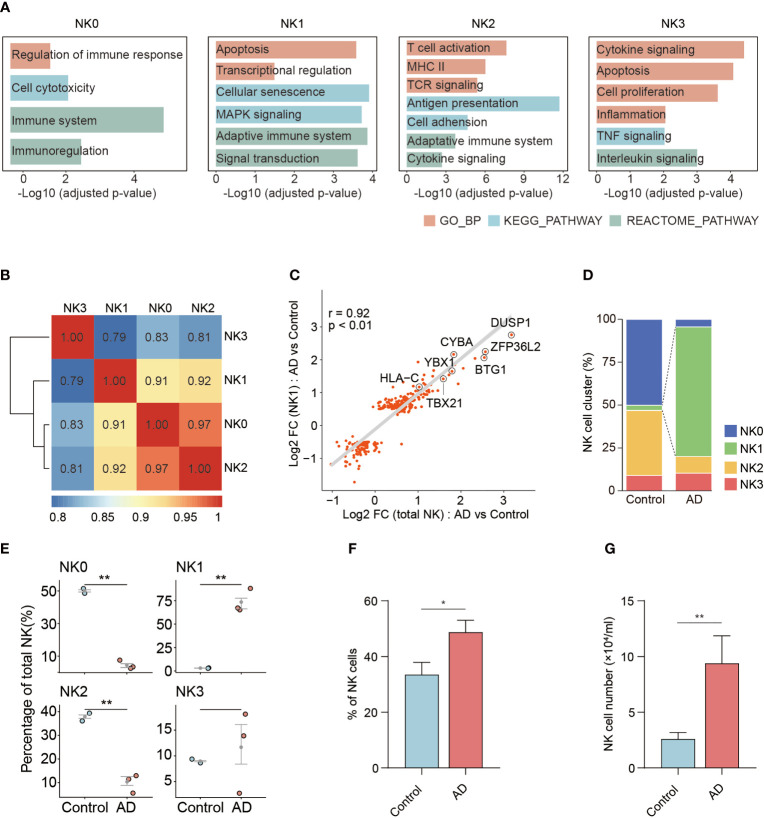
The functional characteristics and distributions of four NK cell subsets. **(A)** Bar plots show the selected GO and KEGG as well as Reactome terms enrichment for each of the four NK cell subsets in patients with AD versus controls. The significance threshold was set to *p* < 0.05. **(B)** Correlations among four NK subsets. **(C)** Scatter plot showing the correlation between total fold changes of NK cells’ gene expression from AD versus control (*x*-axis) against NK1 subset fold changes from AD versus control analysis (*y*-axis). Selected top genes are shown. **(D)** The distribution of four NK subsets in patients with AD and controls. **(E)** The relative proportion of each cluster was calculated in each sample. **(F)** Bar plot showed the CX3CR1 expressing NK cell percent in AD and control groups. **(G)** Bar plot showed the number of CX3CR1 expressing NK cell in groups of AD patients and control subjects. In **(F**, **G)**, AD: *n* = 7, control: *n* = 11. Data are presented as means ± SEM. *p < 0.05, **p < 0.01.

Gene signature module score analysis revealed that the NK0, NK1, and NK2 clusters resemble CD56^dim^ NK cells (13–15), whereas the NK3 cluster resembles CD56^bright^ NK cells (**Figure 3D**). Of note, the NK2 subset shared the highest adaptive NK cell gene set score (**Figure 3D**). GO, KEGG, and Reactome enrichment analysis revealed that the NK0 cluster had an enrichment of cytotoxicity (**Figure 4A**). The NK1 subset was enriched in apoptotic process and cellular senescence, accompanied by upregulation of CX3CR1 and KLF2 (**Figure 4A**). In contrast, NK2 displayed an enrichment in adaptive features of NK cells (**Figure 4A**). The NK3 cluster was enriched in cytokine signaling and pathways related to immune regulatory function (**Figure 4A**).

Correlation analysis was performed to measure the similarity among these four NK cell clusters. As shown in **Figure 4B**, the NK0 cluster was similar to the NK2 cluster. The NK1 cluster had a weak similarity with NK0 and NK2 clusters. In contrast, NK3 represented a distinct subset to other NK cell clusters. Correlation analysis of DEGs revealed the NK1 subset as an enriched subset in AD relative to controls (**Figures 4C, D**, **Supplementary Figure 1F**), accompanied by a contraction of the NK0 subset and the NK2 subset (**Figures 4D, E**).

Similarly, flow cytometry results show that the percentage and number of NK cells expressing CX3CR1 were increased in AD patients (**Figures 4F, G**). A negative correlation was seen between the number of CX3CR1-expressing NK cells and the severity of cognitive impairment (**Supplementary Figure 2E**).

These results demonstrate the expansion of a distinct blood NK cell subset expressing CX3CR1, TBX21, MYOM2, DUSP1, and ZFP36L2 in AD patients and its relation to cognitive impairment.

### Pseudo-temporal ordering of blood NK cells reveals a branched trajectory with a significant shift toward the NK1 subset in patients with AD

**Figure 5:**
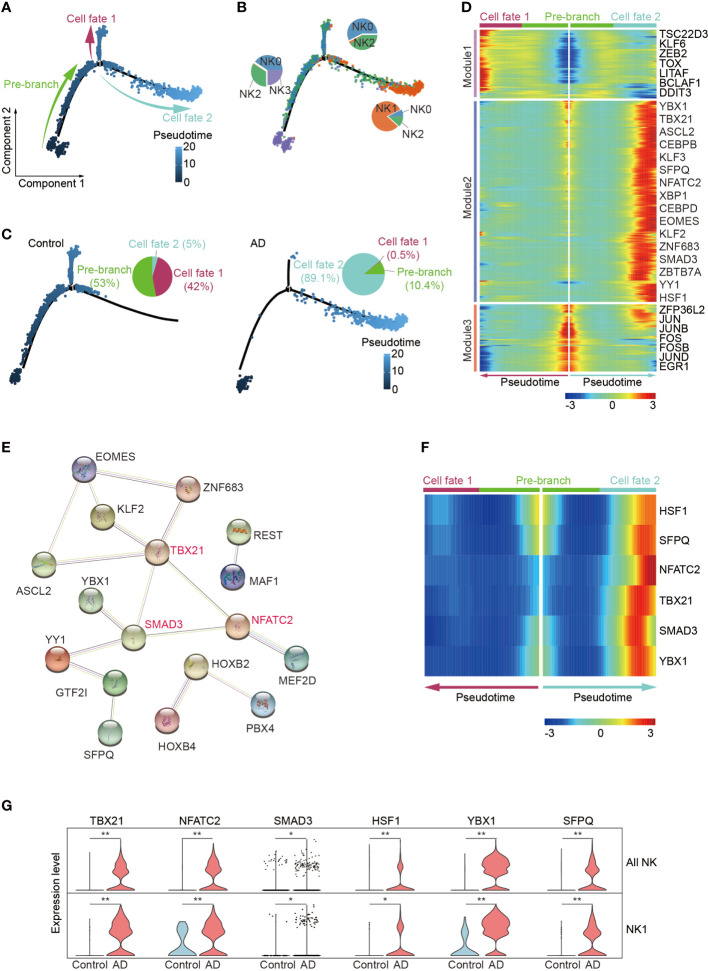
Pseudo-temporal ordering of NK cells reveals a branched trajectory with a significant shift toward the NK1 subset in AD. **(A)** Pseudo-time ordering of NK cells shows a branched trajectory. **(B)** The distribution of the four NK subsets among each branch. **(C)** Evident shift toward a NK1 phenotype (cell fate 2) in AD (right panel) versus control (left panel). **(D)** Hierarchical clustering of the branch-dependent genes reveals three gene modules. The significance threshold was set to a *q*-value of the branched expression analysis modeling test < 1e-04. The transcription factors involved in each module are shown. **(E)** The protein–protein interaction network of the module 2 transcription factors was constructed by STRING. TBX21, SMAD3, and NFATC2 are colored red and serve as hub genes. **(F)** Six key TFs displaying branch dependence are from gene module 2; most of them are enriched in cell fate 2. **(G)** Violin plots show the expression levels of six specific transcription factors in AD and control group. *p < 0.05, **p < 0.01.

Since the enrichment of CX3CR1 and TBX21 in the NK1 subset suggests augmented maturation of NK cells, we next conducted pseudo-temporal analysis with Monocle2. The pseudo-time analysis ordered cells along a trajectory that segregates 2,897 NK cells into two major branches of cell fate 1 and cell fate 2, highlighting a specific developmental trajectory of NK cells. As shown in **Figures 5A, B**, the cell fate 1 branch was mostly constituted by the CD56^dim^ effector NK cell subset and the adaptive NK cell subset, whereas the cell fate 2 branch was mainly constituted by the NK1 subset. CD56^bright^ NK cells were mainly distributed in the top trajectory of the pre-branch that represents the initial state of NK cells. Notably, the trajectory in AD displayed an evident shift toward the NK1 subset in the cell fate 2 branch (AD: 89.1%, Control: 5.0%) (**Figure 5C**).

We identified 682 branch-dependent genes during the cellular state transition from the pre-branch to cell fate 1 and cell fate 2 through branched expression analysis. Hierarchical clustering of these genes revealed three gene modules. Representative TFs for each gene module are shown in **Figure 5D**. Among these modules, most of the genes in module 2 were concentrated in the cell fate 2 branch cells. Notably, TBX21, NFATC2, and SMAD3 are representative TFs of module 2 and may serve as hub genes in control of NK cell alterations in AD (**Figures 5E, F**). Meantime, these genes’ expression level was enriched in patients with AD versus controls (**Figure 5G**).

These results demonstrate that the blood NK1 subset in patients with AD is at the late stage of NK cell development and key TFs including TBX21, NFATC2, and SMAD3 may play a vital role in this subset expansion.

## Discussion

The major goal of this work was to address the alterations of human NK cell transcriptome in AD from an unbiased transcriptome-wide perspective, and identify an NK cell subset that may be linked to disease pathogenesis. As documented here, we found reduced numbers and cytotoxic activity of blood NK cells in AD. Among identified NK cell subsets (i.e., NK0, NK1, NK2, and NK3), a unique NK1 subset is expanded in AD and characterized by expression of CX3CR1, TBX21, MYOM2, DUSP1, and ZFP36L2. Pseudo-time analysis identified that this distinct NK cell subset is at a late stage of NK cell development, accompanied by an increased expression of TFs of TBX21, NFATC2, and SMAD3. Flow cytometry analysis of blood NK cells from AD patients revealed this subset, together with its association with cognitive impairment.

In this study, we subclustered NK cells at single-cell resolution and identified four different subsets. We found reductions of cytotoxic (NK0) and adaptive (NK2) subclusters in the blood of AD patients. Previous single-cell studies have demonstrated the adaptive features of NK cells expressing KLRC2 (NKG2C), CD52, and IL32 (15), along with expression of antigen presentation and T-cell activation markers (14). Consistent with the above studies, we also found adaptive features of NK cells such as the lack of CD3 and the expression of NK cell markers (NKG7, KLRD1, and NCR1). The lower distribution of cytotoxic (NK0) and adaptive (NK2) subsets in the blood suggests that these NK cells may be mobilized and recruited into other organ compartments in AD. Another explanation could be a result from altered neurogenic innervations toward these NK cell subsets, leading to their reduction, although other possibilities cannot be excluded. These postulations await future investigations.

CX3CR1 is involved in the chemotaxis of leukocytes; a previous study revealed a beneficial role of CX3CR1^+^ NK cells in experimental autoimmune encephalomyelitis (EAE), in a mouse model of multiple sclerosis (MS) (18). Another study suggested that CX3CR1 could identify a late stage of NK cell development characterized by decreased effector function (19). In this study, we found reduced NK cell cytotoxicity and expansion in a unique subset of CX3CR1^+^TBX21^+^ NK cells associated with cognitive impairment in AD patients. Trajectory analysis suggests that the expansion of this NK cell subset in AD patients may have resulted from augmented differentiation from the NK3 subcluster, although enriched apoptotic processes were noted in this subcluster. Nevertheless, the discrepancy between previous studies and our findings may involve distinct features of NK cells across different disease conditions, i.e., MS vs. AD, and the potential discrepancies of NK cells in humans vs. mice. Future studies are required to pinpoint NK cell features and their precise contributions to AD progression.

Although a few studies have suggested the involvement of NK cells in AD patients and mouse models (20, 21), it is still early to conclude the precise impact of NK cells on the initiation and progression of AD pathology. NK cells participate in CNS inflammatory injury once they sense danger signals and are receptive to neurogenic innervations in brain disorders (22, 23). It is reasonable to postulate that the alterations of NK cell signatures are likely, at least partially, the result of the brain pathology in AD. On the other hand, the altered features of NK cells may be involved in AD pathology, albeit further studies are required to better understand the role of NK cells during disease progression. Additionally, the small sample size in a Chinese cohort of both single-cell analysis and flow cytometry tests is a limiting factor to interpret our findings. Future studies are required to verify these results among large populations including Chinese and subjects from other countries.

In summary, our study demonstrated the alterations of transcriptomic profile in blood NK cells from patients with AD and identified a distinct NK cell subset related to cognitive decline. These new results provide additional support to the involvement of NK cells in AD pathology.

## Data availability statement

Publicly available datasets were analyzed in this study. This data can be found here: https://www.ncbi.nlm.nih.gov/geo/query/acc.cgi?acc=GSE181279.

## Author contributions

QL, HL, and CQ designed research. NZ, FL, and YH help to collect peripheral blood samples or finish the flow cytometry experiments. CQ, WZ, and HL analyzed data and drafted the manuscript. QL edited the manuscript. All authors read and approved the manuscript.

## Funding

This study was supported in part by the Natural Science Foundation of Tianjin Education Commission (2020KJ148), the Natural Science Foundation of Tianjin (18JCZDJC97600), and the National Natural Science Foundation of China (82101373).

## Acknowledgments

The authors thank all team members in the Department of Neurology and Tianjin Neuroimmunology Institute at Tianjin Medical University General Hospital for their support.

## Conflict of interest

The authors declare that the research was conducted in the absence of any commercial or financial relationships that could be construed as a potential conflict of interest.

## Publisher’s note

All claims expressed in this article are solely those of the authors and do not necessarily represent those of their affiliated organizations, or those of the publisher, the editors and the reviewers. Any product that may be evaluated in this article, or claim that may be made by its manufacturer, is not guaranteed or endorsed by the publisher.
