# Supplementary material for: Alzheimer’s disease alters the transcriptomic profile of natural killer cells at single-cell resolution
Source: Front Immunol. 2022 Nov 2;13:1004885. doi: 10.3389/fimmu.2022.1004885 (PMC9666759; doi:10.3389/fimmu.2022.1004885)
Supplement: Supplementary file 1 [file DataSheet_1.pdf]

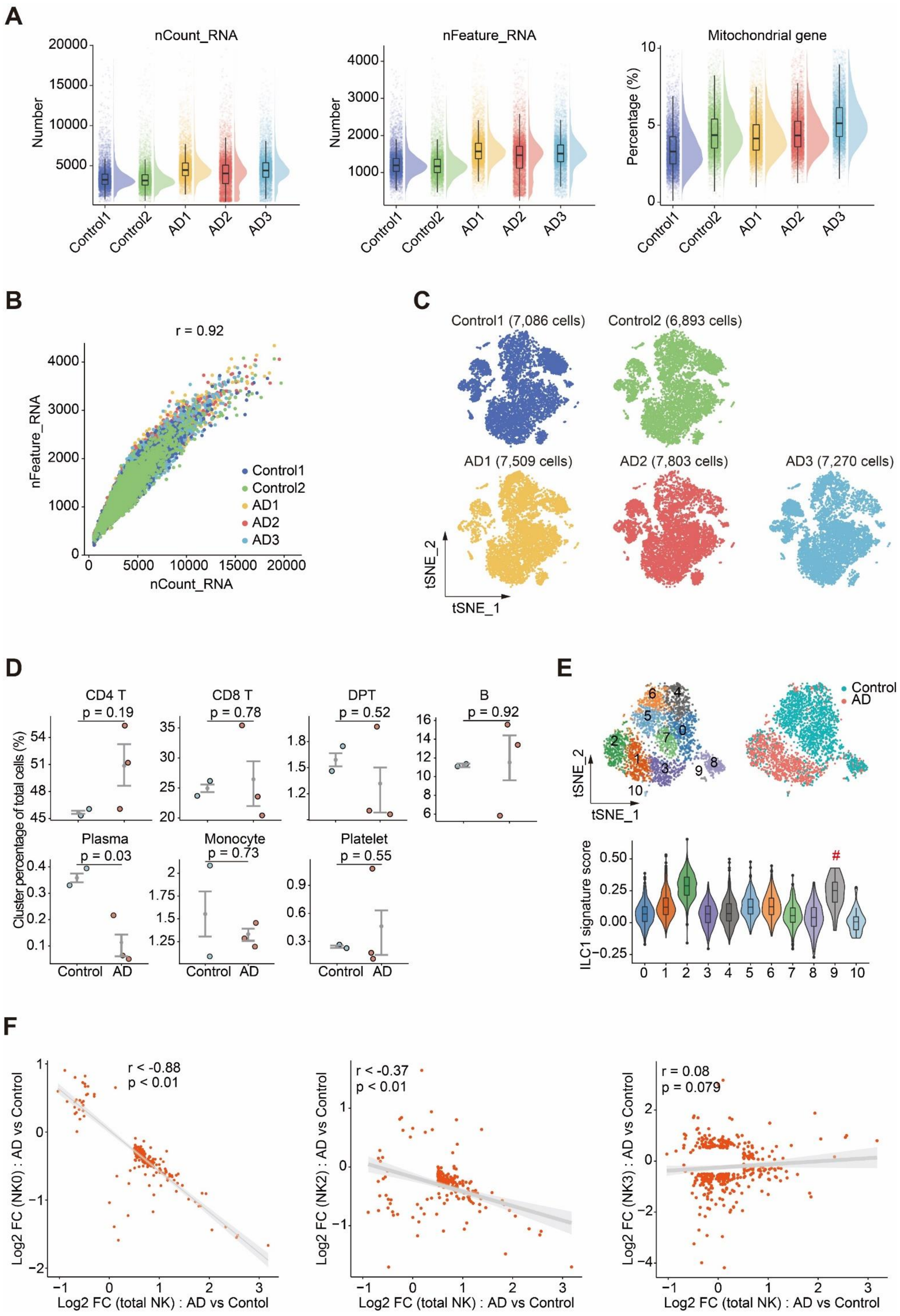

A
nCount_RNA
nFeature_RNA
Mitochondrial gene
Number
Percentage (%)
Control1
Control2
AD1
AD2
AD3
B
r = 0.92
nFeature_RNA
nCount_RNA
C
Control1 (7,086 cells)
Control2 (6,893 cells)
AD1 (7,509 cells)
AD2 (7,803 cells)
AD3 (7,270 cells)
tSNE_2
tSNE_1
D
CD4 T
p = 0.19
CD8 T
p = 0.78
DPT
p = 0.52
B
p = 0.92
Plasma
p = 0.03
Monocyte
p = 0.73
Platelet
p = 0.55
Cluster percentage of total cells (%)
Control
AD
E
Control
AD
ILC1 signature score
#
F
r < -0.88
p < 0.01
r < -0.37
p < 0.01
r = 0.08
p = 0.079
Log2 FC (NK0) : AD vs Control
Log2 FC (NK2) : AD vs Control
Log2 FC (NK3) : AD vs Control
Log2 FC (total NK) : AD vs Contol
Log2 FC (total NK) : AD vs Control

Supplementary Figure 1. Single cell analysis of NK cells and ILC1 in blood PBMCs from patients with AD.

(**A**) The counts of UMI (nCount_RNA), genes (nFeatutre_RNA) and mitochondrial gene fraction in each sample after data quality control. (**B**) The correlation between genes and UMI of each sample. (**C**) tSNE plots of 36,561 single cells from two control subjects and three AD patients, colored according to sample origins. **(D)** The distribution of CD4 T, CD8 T, DPT, B, plasma, monocyte and platelet subsets in AD and control groups, Data are presented as means ± SEM. * $p < 0.05$, ** $p < 0.01$. **(E)** tSNE plots of 3,361 single NK cells, colored by cell clusters and group; Violin plot displayed ILC1 signatures score assessed by AddModuleScore function, the cluster 2 and 9 have higher score and were removed for further analyses. (**F**) Scatterplot comparing total NK cells gene expression fold changes from AD versus control analysis (x axis) against the AD-specific NK0, NK2 and NK3 subset fold changes from AD versus control analysis (y axis).

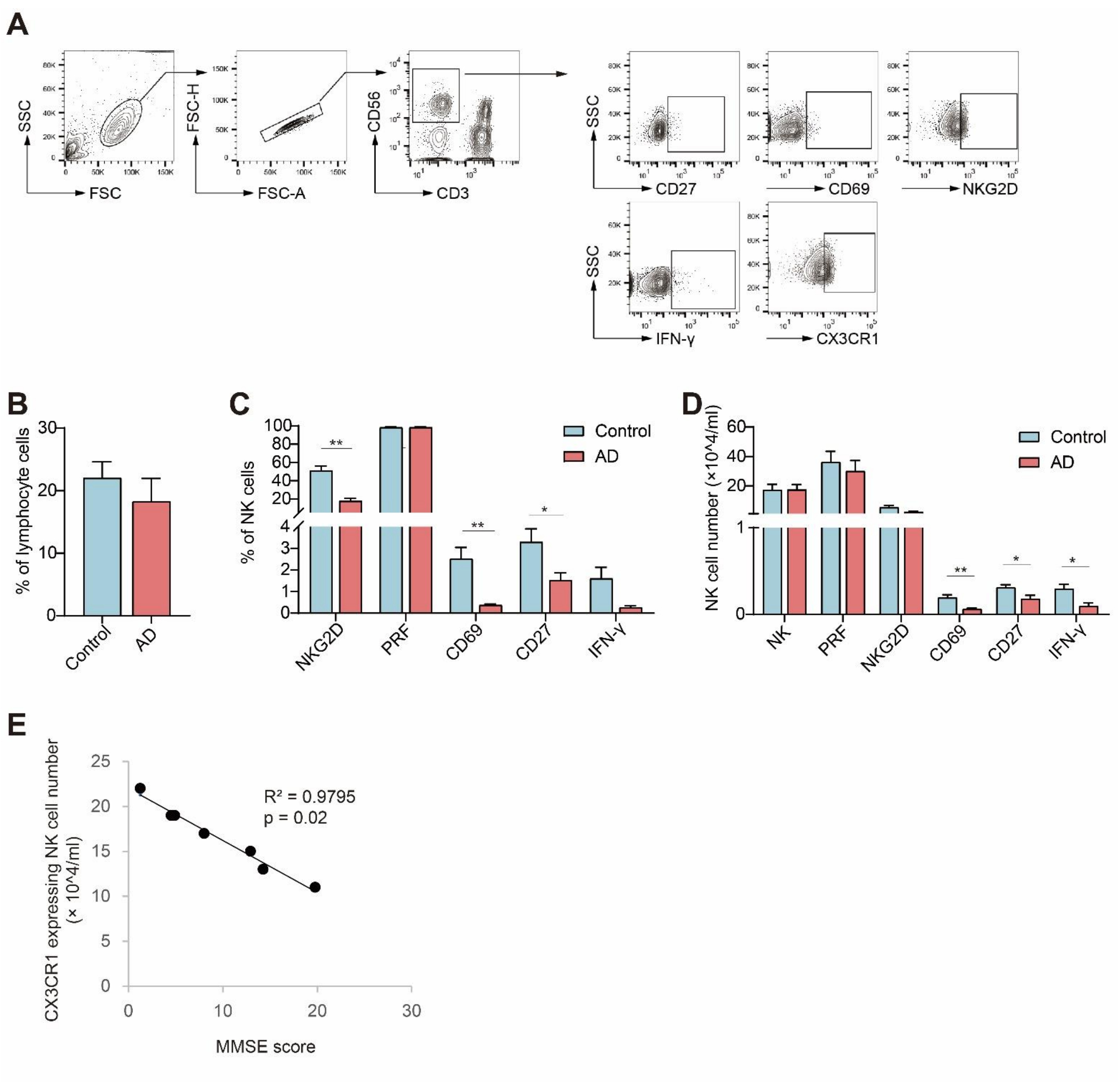

Supplementary Figure 2. Reduced NK cell number and activity in AD, peripheral blood were obtained from 7 patients with AD and 11 control subjects.

(**A**) Flow cytometry plots showing the expression of NK cell activating receptors (CD69, CD27), cytotoxicity receptors (NKG2D), and cytokine (IFN-γ). (**B**) Bar graph showing the percentage of NK cells from groups of AD patients and control subjects. (**C**) Bar graph showing the percentage of NK cells expressing NKG2D, PRF, CD69, CD27 and IFN-γ in indicated groups. (**D**) Bar graph showing the absolute number of NK cells and NK cells that express NKG2D, PRF, CD69, CD27 and IFN-γ in indicated groups. (**E**) Correlation between the number of CX3CR1-expressing NK cells and MMSE score in AD patients. In **B-E**, AD: n = 7,

control: n = 11. Data are presented as means ± SEM. $^{*}p < 0.05$, $^{**}p < 0.01$.
